# The influence of Al^3+^ on DNA methylation and sequence changes in the triticale (× *Triticosecale* Wittmack) genome

**DOI:** 10.1007/s13353-018-0459-0

**Published:** 2018-08-30

**Authors:** Agnieszka Niedziela

**Affiliations:** Department of Plant Physiology and Biochemistry, Plant Breeding and Acclimatization Institute, National Research Institute, 05-870 Radzików, Błonie Poland

**Keywords:** Epigenetics, Methylation, Sequence variation, Aluminum stress, Triticale

## Abstract

Abiotic stressors such as drought, salinity, and exposure to heavy metals can induce epigenetic changes in plants. In this study, liquid chromatography (RP-HPLC), methylation amplified fragment length polymorphisms (metAFLP), and methylation-sensitive amplification polymorphisms (MSAP) analysis was used to investigate the effects of aluminum (Al) stress on DNA methylation levels in the crop species triticale. RP-HPLC, but not metAFLP or MSAP, revealed significant differences in methylation between Al-tolerant (T) and non-tolerant (NT) triticale lines. The direction of methylation change was dependent on phenotype and organ. Al treatment increased the level of global DNA methylation in roots of T lines by approximately 0.6%, whereas demethylation of approximately 1.0% was observed in NT lines. DNA methylation in leaves was not affected by Al stress. The metAFLP and MSAP approaches identified DNA alterations induced by Al^3+^ treatment. The metAFLP technique revealed sequence changes in roots of all analyzed triticale lines and few mutations in leaves. MSAP showed that demethylation of CCGG sites reached approximately 3.97% and 3.75% for T and NT lines, respectively, and was more abundant than de novo methylation, which was observed only in two tolerant lines affected by Al stress. Three of the MSAP fragments showed similarity to genes involved in abiotic stress.

**Electronic supplementary material:**

The online version of this article (10.1007/s13353-018-0459-0) contains supplementary material, which is available to authorized users.

## Introduction

Plants growing under stressful conditions may experience genetic and/or DNA methylation pattern changes. For example, a *Poa annua* population under Antarctic climatic conditions differed from its founding European counterpart mostly at the DNA methylation level, while sequence changes were relatively small (Chwedorzewska and Bednarek 2012). In some plant species, alterations to DNA methylation patterns occurred in response to exposure to drought (Wang et al. 2011; Tang et al. 2014), cold (Pan et al. 2011), salt (Cao et al. 2011; Karan et al. 2012), or heavy metals in soil (Erturk et al. 2015).

Abiotic stress may result in either DNA demethylation or de novo methylation (Sahu et al. 2013). The direction of the methylation change depends on a range of factors such as species (Aina et al. 2004), phenotype (tolerant/non-tolerant) (Pan et al. 2011; Karan et al. 2012), developmental stage (Wang et al. 2011), organ type (Karan et al. 2012), and abiotic stress severity (Zhong et al. 2009; Greco et al. 2012). In barley (Bednarek et al. 2007) and *Gentiana pannonica* (Fiuk et al. 2010), in vitro tissue culture increased de novo methylation in the regenerated plants. By contrast, triticale regenerants derived from in vitro tissue cultures experienced DNA demethylation (Machczyńska et al. 2014a, b). Similarly, exposure to different metals can affect the direction of DNA methylation change. For example, cadmium promoted cytosine methylation in *Posidonia oceanica* (Greco et al. 2012), while its presence together with nickel and chromium decreased DNA methylation in roots of clover and hemp (Aina et al. 2004). Marconi et al. (2013) revealed that salt stress decreased the percentage of 5-methylcytosine (m5C) in a tolerant rapeseed genotype but increased it in a non-tolerant genotype. Similarly, in drought-stressed rice (Gayacharan and Joel 2013), tolerant genotypes exhibited demethylation (up to 11.1%), whereas non-tolerant genotypes experience de novo methylation (up to 36%). Nevertheless, methylation change is not always associated with plant tolerance to stress. Karan et al. (2012) documented that salinity increased the m5C level in roots of stressed rice, and the direction of change was the same for tolerant (Pokkali and Geumgangbyeo) and non-tolerant (IR29 and Nipponbare) genotypes. However, in shoots, salinity promoted methylation in non-tolerant Nipponbare and tolerant Pokkali and induced a decrease in methylation in the other two genotypes, IR29 and Geumgangbyeo, which were salt-intolerant and salt-tolerant, respectively.

Aluminum (Al) stress frequently occurs in plants growing in soils under acidic conditions (Kochian 1995). Several genes belonging to the aluminum-activated malate transporter (ALMT), ATP-binding cassette transporter (ABC), multidrug and toxin efflux (MATE), and Cys2-His2-type zinc-finger transcription factor (sensitive to proton rhizotoxicity [STOP1] and Al resistance transcription factor1 [ART1]) families are involved in responding to Al stress (Sasaki et al. 2004; Yamaji et al. 2009; Huang et al. 2010; Maron et al. 2010; Garcia-Oliveira et al. 2013). Depending on species, these genes explain up to 60% of the phenotypic variance (Nguyen et al. 2002; Hoekenga et al. 2003; Maron et al. 2010; Silva-Navas et al. 2012). In triticale, QTLs associated with Al tolerance were located on chromosomes 3R, 4R, 6R, and 7R (Niedziela et al. 2012). Despite the multigenic nature of this response, the ALMT QTL that explained 36% of phenotypic variance appeared to be the major trait determinant (Niedziela et al. 2014). However, the extent of the changes evoked by the presence of Al in soil and their impact on epigenetic changes to DNA methylation remain unclear. To date, no systematic studies have examined the epigenetic aspects of Al tolerance in triticale. Our preliminary study involving semi-quantitative examination of methylation-sensitive amplification polymorphisms (MSAPs) (Bednarek et al. 2017) did not fully explore changes to methylation in response to Al stress.

Many techniques have been developed for the estimation of 5mC content (Yong et al. 2016), either at a genome-wide level or at specific sequences. High-performance liquid chromatography (HPLC) is considered the most reliable approach to study genome-wide cytosine methylation (Johnston et al. 2005). However, possibly as a result of the robustness of the approach, relatively few studies have been published using HPLC to examine abiotic stress (Choi and Sano 2007; Barientos et al. 2013; Machczyńska et al. 2014a; Wang et al. 2014). Alternatives to HPLC for genome-wide analysis of methylation include the recently developed methylation amplified fragment length polymorphisms (metAFLP) technique (Bednarek et al. 2007) and the well-established MSAP approach (Reyna-López et al. 1997). Both techniques employ restriction enzymes with the same recognition sequence that is sensitive or insensitive to methylation status.

The study examined whether Al tolerance in triticale was affected by DNA methylation differences between tolerant and non-tolerant inbred lines using HPLC, metAFLP, and MSAP approaches. This study may provide broader insights into problematic traits modified by environmental factors.

## Material and methods

### Plant material and growing conditions

Triticale inbred lines were provided by the Małyszyn Experimental Station (Poland). Lines were selected based on their tolerance to Al stress (Niedziela et al. 2012). Five Al-tolerant (T; L195 [MAH32969-15 × Dublet], L198 [MAH3405(Milewo) × Matejko], L201 [MAH27470-1 × Bacum], L210 [MAH2601(Matejko) × Caracal], L451 [MAH25163-2 × DED244/98]) and five non-tolerant (NT; L203 [MAH27470-1 × Bacum], L438 [MAH3198 × DED1231/97], L444 [MAH3198 × CHD2807/98-7-1], L455 [B-123 USA × Witon], L461 [NORD7153 × Moderato]) triticale lines were exposed to Al stress using the physiological test described by Anioł (1984). The test was repeated several times in previous experiments to select successive generations of chosen inbred lines up to generation S10 (not presented). Seeds (approximately 100 per line) were sterilized with 10% sodium hypochlorite for 10 min, rinsed thoroughly with water, and germinated overnight on filter paper in Petri dishes. Sprouted seeds representative of each inbred line were sown on two individual polyethylene nets fixed in plastic frames (50 seeds per net; two nets per line) and protected from sinking with polystyrene foam. The nets were floated in a tray filled with aerated nutrient solution (Anioł 1984). Seedlings were maintained in a controlled-environment growth cabinet (POL-EKO-APARATURA, ST500 B40 FOT10) with conditions of temperature 25 °C and photoperiod 12/12 h day/night. After 3 days, one of the two polyethylene nets for each inbred line was transferred to the same nutrient medium supplied with 15, 20, or 30 ppm Al^3+^ as AlCl_3_. The nutrient solution with Al^3+^ was maintained at pH 4.5, adjusted with 0.1-M HCl. The second polyethylene net for each inbred line was maintained under control conditions (without Al) throughout the experiment. After 24 h of incubation in the medium containing Al, seedlings were thoroughly washed for 2–3 min in running water and then transferred to the nutrient solution without Al for 48 h. Seedlings were then removed from the control and Al stress nutrient solutions, and root regrowth of Al-treated seedlings was evaluated. Leaves (3 cm from the top parts) and root tips (3–4 mm long) from 7-day-old seedlings were used for genomic DNA isolation. Eriochrome cyanine R staining for visualization of Al-damaged regions, as described by Anioł (1984), was not performed. Nevertheless, areas affected by Al were easily recognized due to their light yellow coloration.

### DNA isolation

Total genomic DNA was isolated from pooled leaves and root tips collected from control (C) and Al-stressed (S) seedlings using a Plant DNeasyMiniKit (Qiagen, Hilden, Germany). DNA quantity was assessed spectrophotometrically, and integrity and purity were checked with 1.0% agarose gel electrophoresis. DNA samples extracted from the same seedling pools were used for all experiments.

### Reversed-phase HPLC (RP-HPLC)

DNA samples (5 μg) were denatured by heating to 100 °C and then hydrolyzed for 17 h at 37 °C in a solution containing 5 μl of 10-mM ZnSO_4_ and 10 μl of 1.0 U ml^−1^ P1 nuclease in 30-mM NaOAc (pH 5.4). After nuclease digestion, 10 μl of 0.5-M tris pH 8.3 and 10 μl of 10 U ml^−1^ alkaline phosphatase in 2.5-M (NH_4_)_2_SO_4_ were added, followed by incubation at 37 °C for 2 h, then centrifugation at 12,000 rpm for 5 min.

Reversed-phase HPLC (RP-HPLC) quantification of overall DNA methylation was performed as described by Johnston et al. (2005) using a Waters 625 LC system connected to a Millennium 32v 4.0 data processing station. Briefly, a 4u Max-RP C12 (250 9 4.6 mm, Phenomenex) column combined with a 4u Max-RP C12 pre-column was used with a linear gradient of eluent. Two eluents were used: eluent A, 0.5% methanol in 10-mM KH_2_PO_4_ (*v*/v); and eluent B, 10% methanol in 10-mM KH_2_PO_4_. A linear gradient of 10 min 100% A and 100% B, and 15 min 100% B and 100% A, with 5 min of total running time, was used. The percentage of deoxycytidine methylation in relation to the total content of cytidine was calculated according to the following equation: 5mdC% = [5mdC/ (5mdC + dC)] × 100, where 5mdC and dC represent 5-methyldeoxycytidine and deoxycytidine, respectively. Three RP-HPLC technical replicates were conducted for each DNA sample.

### Methylation amplified fragment length polymorphism analysis

Genomic DNA (0.5 μg) for metAFLP analysis was subjected to two separate digestions with *Acc*65I/*Mse*I and *Kpn*I/*Mse*I endonucleases. Reactions were performed in 15-μl volumes containing 10-U *Mse*I, 3.0-U *Acc*65I or *Kpn*I, 1× ligation buffer, 50-nM NaCl, and 0.5-mg/ml BSA. The *Acc65*I and *Mse*I neoschizomers, *Mse*I endonuclease, BSA, and ligation buffer were purchased from New England Biolabs, Beverly, MA, USA. Digestions were incubated for 3 h at 37 °C followed by 15 min at 70 °C. After enzymatic digestion of the DNA samples, adaptors were ligated to the released ends. The ligation mixture (25 μl) consisted of 1× ligation buffer, 50-mM NaCl, 0.5-mg/ml BSA, 1.5-pM *Mse*I adapters, 0.15-pM *Acc65*I (or *Kpn*I) adapters, and 120-U T4 DNA ligase (New England Biolabs). The reaction was run for 12 h at 20 °C following dilution (1:3 in MiliQ H_2_O). A pre-amplification step was conducted in a final volume of 25 μl (2.5 μl of diluted ligation mixture, 40 pM of each preselective primer, 1× PCR buffer [Qiagen], 2.5-mM MgCl_2_, 0.4-mM dNTPs, and 0.5-U Taq polymerase) using 20 cycles of 94 °C for 30 s, 56 °C for 30 s, and 72 °C for 1 min. The pre-amplified products were then diluted 1:20 (PCR product, MiliQ H_2_O). Selective amplification was conducted in a final volume of 10 μl (1.5 μl of the pre-amplification product, 0.5-pM *Mse*I selective primer, 0.15-pM *Acc65*I/*Kpn*I [5′-^32^P] end-labeled primers, 1× PCR buffer [Qiagen], 2.5-mM MgCl_2_, 0.4-mM dNTPs, and 0.0125-U HotStart DNA polymerase [Qiagen]). Fifteen primer pairs were used (Table S1). The following thermal profile was used: 95 °C, 15 min; 12 × (94 °C, 30 s; 65 °C ramp, 0.7 °C, 30 s; 72 °C, 60 s); 29 × (94 °C, 30 s; 56 °C, 30 s; 72 °C, 60 s); 72 °C, 10 min; 5 °C, ∞. Denatured PCR products were separated on a 7% denaturing polyacrylamide gel and exposed to X-ray film overnight at − 70 °C. All metAFLP analysis was performed twice using DNA samples isolated from the same lines.

### Methylation-sensitive amplification polymorphisms profiling

The MSAP procedure was based on the metAFLP approach, with *Hpa*II and *Msp*I endonucleases used instead of *Acc*65I and *Kpn*I, as described by Xiong et al. (1999). Genomic DNA samples were digested with *Hpa*II/*Eco*RI and *Msp*I/*Eco*RI endonucleases (New England Biolabs). Digestion mixes contained 5.0-U *Hpa*II, 5.0-U *Eco*RI, 1× ligation buffer, 50-mM NaCl, and 0.5-mg/ml BSA. Digestion, adapter ligation, and preselective and selective PCR were performed as described for metAFLP. Denatured PCR products were separated on a 7.0% denaturing polyacrylamide gel and exposed to X-ray film overnight at − 70 °C. The sequences of *Eco*RI and *Hpa*II/*Msp*I adapters and preselective and selective primers were as described by Xiong et al. (1999). Fourteen selective primer pair combinations with three selective nucleotides for the *Eco*RI ends, and three or four selective nucleotides for the *Hpa*II-*Msp*I ends, were used in the experiment (Table S2). MSAP analysis was repeated twice using DNA samples isolated from the same lines.

### Sequencing

Differentially amplified MSAP fragments were recovered from the gel, re-amplified, and purified using a QIAquick PCR purification kit (Qiagen). Sequencing was performed using a SequiTerm Excel™ II DNA sequencing kit (Epicenter, Madison, WI, USA) with respective end-radiolabeled [5′-^32^P] MSAP primers according to the manufacturer’s instructions. Sequences were analyzed visually.

### Bioinformatics

BLASTN searches were conducted against the non-redundant database (NR) in NCBI against the Gramineae family.

## Molecular data analysis

### Methylation amplified fragment length polymorphisms

The metAFLP profiles generated by the *Acc65*I/*Mse*I and *Kpn*I/*Mse*I digests were converted into four-digit binary code. The first and third positions of the binary code indicated the presence (1) or absence (0) of a marker in the profile of the non-stressed plant (C, control) digested with *Acc65*I/*Mse*I and *Kpn*I/*Mse*I, respectively. The second and fourth positions reflected the same situation but for the stressed (S) plant. Sixteen possible four-digit binary codes were grouped into various event types: sequence (SE), demethylation (DME), de novo methylation (DNME), and complex (CE) events (Bednarek et al. 2007) and used for the estimation of sequence (SV), demethylation (DMV), de novo methylation (DNMV), and complex (CV) variation, expressed in percentages. All types of variation taken together described total abiotic stress-induced variation (ASIV). The percentages of the global genome restriction sites that were methylated (GM) and non-methylated (GNM) in Al-stressed plants were also calculated.

### Methylation-sensitive amplification polymorphisms

MSAP patterns were sorted into four types according to their methylation status, as described by Wang et al. (2011). Accordingly, the presence of MSAP bands generated by *Hpa*II/*Eco*RI and *Msp*I/*Eco*RI digests (encoded as 11), simultaneously indicating non-methylated sites, was classified as Type I. Type II fragments were those present only in *Hpa*II/*Eco*RI (10) digests, whereas those present in *Msp*I/*Eco*RI (01) digests were labeled as Type III. The absence of a band for both enzyme combinations (00) was assigned to Type IV. Type II patterns reflected hemi-methylation, whereas Types III and IV indicated fully methylated restriction sites.

The total methylated status (TMS) of the restriction sites was expressed using percentages and calculated using the sum of patterns of the given type according to the formula TMS = [(II + III + IV) / (I + II + III + IV)] × 100%. Similarly, the number of fully methylated sites (FM) was estimated as follows: FM = [(III + IV) / (I + II + III + IV)] × 100%. Hemi-methylation (HM) and non-methylation (NM) quantitative characteristics were determined using HM = [(II) / (I + II + III + IV)] × 100% and NM = [(I) / (I + II + III + IV)] × 100%, respectively. Such calculations were carried out for each genotype.

To evaluate the changes in methylation pattern resulting from Al stress, MSAP profile types were compared for each given marker between the same C and S inbred lines. Transitions encoded as I-I (following classification as Type I, II, III, or IV), II-II, III-III, and IV-IV indicated no change in methylation status (LCMS). Similarly, I-II, I-III, II-III, I-IV, II-IV, and III-IV transitions were assigned to de novo methylation (DNM). Transitions II-I, III-I, IV-I, III-II, IV-II, and IV-III were assigned to demethylation (DM). The percentages of each transition type were evaluated as follows: LCMS% = 100 × LCMS / (LCMS + DNM + DM); DNM% = 100 × DNM / (LCMS + DNM + DM); and DM% = 100 × DM / (LCMS + DNM + DM).

### Statistical analysis

One-way analysis of variance (ANOVA) with Tukey’s contrast was performed in Statistica 12 (StatSoft. Inc. 2014) at α = 0.05.

## Results

The inbred lines used in this experiment were tested for Al tolerance over several years. All lines demonstrated stable inheritance of the trait. Root regrowth after Al exposure was recorded only in the case of T lines (L195, L198, L201, L210, and L451), with maximum regrowth of 2.8, 1.7, and 0.8 cm for plants treated with 15, 20 and 30 ppm Al^3+^, respectively. NT lines (L203, L438, L444, L455, and L461) failed to grow under Al treatment in a liquid medium.

### Estimation of global methylation by RP-HPLC

RP-HPLC analysis estimated total genomic DNA methylation of T and NT triticale genotypes grown under control conditions to be 22.08–22.71% and 22.95–23.90% for root and leaves, respectively (Tables 1 and 2) depending on the inbred line used.

**Table 1:** RP-HPLC analysis of methylation level (%) in roots of five tolerant (T) and five non-tolerant (NT) triticale lines after exposure to different Al^3+^ concentrations

Line		C (0 ppm)	S (15 ppm)	S (20 ppm)	S (30 ppm)	Δ (S-C) (15 ppm)	Δ (S-C) (20 ppm)	Δ (S-C) (30 ppm)
L438	NT	22.71	21.68	21.71	21.71	− 1.03	− 0.99	− 0.99
L444	NT	22.43	21.21	21.66	20.72	− 1.22	− 0.77	− 1.71
L455	NT	22.53	21.67	21.63	21.58	− 0.86	− 0.98	− 0.95
L461	NT	22.57	21.45	21.62	21.53	− 1.12	− 0.95	− 1.04
L203	NT	22.57	21.75	21.64	NA	− 0.82	− 0.93	NA
Mean (NT lines)		22.56	21.55	21.65	21.39	− 1.01	− 0.91	− 1.17
Tukey’s grouping C, S, and Δ (S-C)		a	b	b	b	a	a	a
L195	T	22.70	23.18	23.35	NA	0.48	0.64	NA
L198	T	22.23	22.97	22.76	22.93	0.74	0.53	0.71
L201	T	22.08	22.56	23.04	22.72	0.50	0.95	0.64
L210	T	22.57	23.35	23.33	23.16	0.78	0.76	0.60
L451	T	22.13	22.62	22.66	22.75	0.49	0.52	0.61
Mean (T lines)		22.35	22.94	23.03	22.89	0.62	0.68	0.64
Tukey’s grouping C, S, and Δ (S-C)		a	b	b	b	b	b	b

One-way analysis of variance (ANOVA) with Tukey’s contrasts (α = 0.05) of DNA methylation changes in three replicate experiments. Mean values (%) that do not share a letter are significantly different. C and S represent control and stressed conditions, respectively. Δ (S-C) reflects the methylation difference between C and S

*NA*, not analyzed

**Table 2:** RP-HPLC analysis of methylation level (%) in leaves of five tolerant (T) and five non-tolerant (NT) triticale lines after exposure to different Al^3+^ concentrations

Line		C (0 ppm)	S (15 ppm)	S (20 ppm)	S (30 ppm)	Δ (S-C) (15 ppm)	Δ (S-C) (20 ppm)	Δ (S-C) (30 ppm)
L438	NT	23.55	23.65	23.15	23.63	0.09	− 0.40	0.08
L444	NT	23.37	23.07	23.75	23.56	− 0.29	0.38	0.19
L455	NT	23.37	23.36	23.32	23.19	− 0.01	− 0.04	− 0.17
L461	NT	23.15	23.35	23.26	23.22	0.20	0.11	0.06
L203	NT	23.90	23.83	24.02	NA	− 0.07	0.12	NA
ANOVA (NT lines)		23.47	23.45	23.53	23.41	− 0.02	0.03	0.04
Tukey’s grouping C, S, and Δ (S-C)		a	a	a	a	a	a	a
L195	T	22.95	23.26	23.25	NA	0.31	0.30	NA
L198	T	23.85	24.13	24.17	24.23	0.29	0.32	0.39
L201	T	23.67	23.87	24.02	23.87	0.20	0.41	0.19
L210	T	23.26	23.36	23.29	23.42	0.10	0.02	0.15
L451	T	23.61	23.77	23.78	23.90	0.16	0.17	0.28
ANOVA (T lines)		23.47	23.67	23.72	23.85	0.21	0.24	0.25
Tukey’s grouping C, S, and Δ (S-C)		a	a	a	a	a	a	a

One-way analysis of variance (ANOVA) with Tukey’s contrasts (α = 0.05) of DNA methylation changes in three replicate experiments. Mean values (%) that do not share a letter are significantly different. C and S represent control and stressed conditions, respectively. Δ (S-C) reflects the methylation difference between C and S

*NA*, not analyzed

ANOVA indicated that 5mdC levels were approximately 1.0% lower in roots of NT lines under stress conditions compared with those under control conditions (Table 1) regardless of Al^3+^ concentration (*F* = 96.24, *p* < 0.001; *F* = 305.4, *p* < 0.001; *F* = 23.58, *p* < 0.001 for 15, 20, and 30 ppm Al^3+^, respectively). DNA methylation levels in roots of T lines exposed to 15, 20, and 30 ppm Al^3+^ increased by approximately 0.65% (*F* = 8.02, *p* < 0.01; *F* = 7.89, *p* < 0.01; *F* = 22.8, *p* < 0.001, respectively) compared with control plants not exposed to stress. No statistically significant differences in methylation were observed in leaves of lines grown under stress and control conditions regardless of Al-tolerance genotype (Table 2).

### Molecular analysis

Preliminary screening of two T and two NT triticale lines using MSAP and metAFLP utilizing 14 and 15 selective primer pairs, respectively, showed identical molecular profiles between lines treated with different concentrations of Al^3+^ (see Fig. S1). Therefore, plants stressed in the presence of 20 ppm Al^3+^ were used for further molecular analysis.

**Fig. 1:**
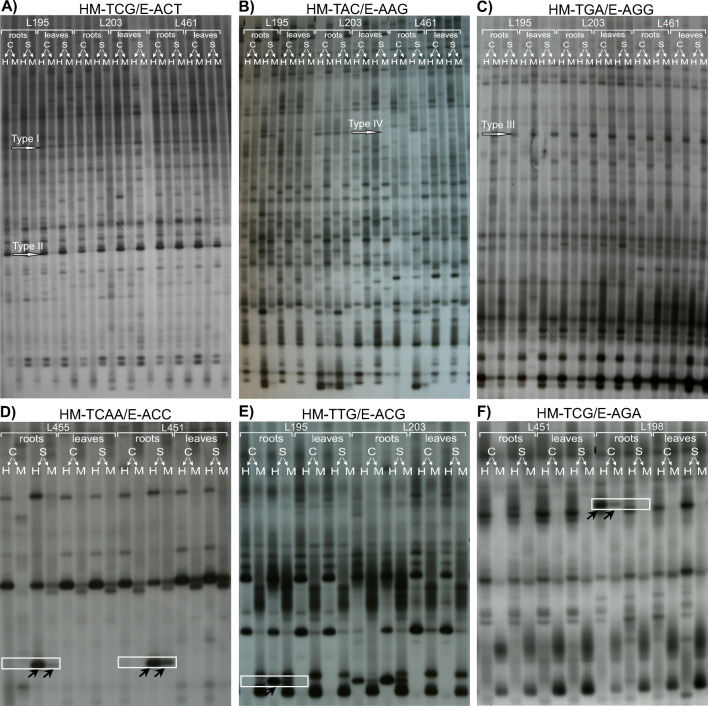
Methylation patterns in roots and leaves of triticale lines (L195, L203, and L461) using the following primer combinations: HM-TCG/E-ACT (**a**), HM-TAC/E-AAG (**b**), and HM-TCG/E-ACT (**c**). *H*, *Hpa*II/*Eco*RI digestion; *M*, *Msp*I/*Eco*RI digestion; *C*, control; and *S*, aluminum treatment (20 ppm). Arrows indicate methylation-sensitive amplification polymorphism (MSAP) bands of unmethylated sites (Type I), hemi-methylated sites (Type II), and fully methylated sites (Types III and IV). Examples of methylation pattern changes (in frames) due to aluminum stress: demethylation of CCGG sites reflected by IV-I (**d**) and IV-II - (**e**) transitions (arrows indicate presence of DNA fragment); de novo methylation reflected by ‘I-IV’ transition (**f**)

The MSAP approach amplified approximately 438 evident and reproducible fragments, including 355 and 361 polymorphic fragments (Type II + III bands), from DNA from roots of NT and T lines under control and stressed conditions, respectively (Table 3; for examples of MSAP gels, see Fig. 1a–c). Slightly more (*F* = 20.52, *p* < 0.001) total methylated bands (Type II + III + IV bands) were evaluated for control conditions (370) than for stressed conditions (361). Under control conditions, the total methylation of CCGG sequences was approximately 84%, with 36% of sites fully methylated (Type III + IV bands) and 49% hemi-methylated (Type II bands) (Table 3, Fig. 1a–c). Under Al stress conditions, approximately 82% of sites were methylated, with 32% being fully methylated and 50% hemi-methylated (Table 3). For leaves, 336 fragments were amplified regardless of control or stress conditions, and 81% of fragments were methylated (33% fully and 48% hemi-methylated) (Table 3).

**Table 3:** Different types of MSAP cytosine methylation levels under aluminum stress and control

Patterns		MSAP band types	Root				Leaf			
			Non-tolerant lines		Tolerant lines		Non-tolerant lines		Tolerant lines	
*Hpa*II	*Msp*I		C	S	C	S	C	S	C	S
1	1	I	67.2^a^	76.0^b^	67.2^a^	76.4^b^	64^a^	64^a^	63.8^a^	63.8^a^
1	0	II	213^a^	219.4^b^	213.6^a^	219.6^b^	160.8^a^	160.8^a^	160.6^a^	160.6^a^
0	1	III	142.2^a^	142.2^a^	142^a^	142^a^	112^a^	112^a^	112^a^	112^a^
0	0	IV	15^a^	0^b^	15.6^a^	0^b^	0^a^	0^a^	0^a^	0^a^
Total amplified bands			437.6^a^	437.6^a^	438.4^a^	438^a^	336.8^a^	336.8^a^	336.4^a^	336.4^a^
Total methylated bands			370.4^a^	361.6^b^	371.2^a^	361.6^b^	272.8^a^	272.8^a^	272.6^a^	272.6^a^
Fully methylated bands			157.2^a^	142.2^b^	157.6^a^	142^b^	112^a^	112^a^	112^a^	112^a^
Total methylated ratio (%)			84.6^a^	82.6^b^	84.7^a^	82.6^b^	80.9^a^	80.9^a^	81.0^a^	81.0^a^
Fully methylated ratio (%)			35.9^a^	32.5^b^	36.0^a^	32.4^b^	33.3^a^	33.3^a^	33.3^a^	33.3^a^
Hemi-methylated ratio (%)			48.7^a^	50.1^a^	48.7^a^	50.1^a^	47.7^a^	47.7^a^	47.7^a^	47.7^a^
Non-methylated ratio (%)			15.4^a^	17.4^b^	15.3^a^	17.4^b^	19.0^a^	19.0^a^	18.9^a^	18.9^a^

One-way analysis of variance (ANOVA) with Tukey’s contrasts (α = 0.05) was applied. Means appended with different letters are significantly different

*C*, control conditions; *S*, plants treated by aluminum (20 ppm)

A score of 1 or 0 represents the presence or absence of bands, respectively

Total methylated ratio = [(II + III + IV) / (I + II + III + IV)] × 100%

Fully methylated ratio = [(III + IV) / (I + II + III + IV)] × 100%

Hemi-methylated ratio = [(II) / (I + II + III + IV)] × 100%

Non-methylated ratio = [(I) / (I + II + III + IV)] × 100%

Type I is unmethylated bands; Type II is hemi-methylated bands; and types III + IV are fully methylated bands. Total methylated bands = II + III + IV

Seven different banding patterns distinguishing the control and Al stress conditions were observed in MSAP gels (Table 4). The transitions I-II, II-II, and III-III, reflecting the lack of changes in banding patterns between control and stressed root materials, were observed in 15.36%, 48.45%, and 32.45% of NT lines, respectively (Table 4). Comparable results for T lines equaled 15.28%, 48.29%, and 32.38%, respectively (Table 4). Differences between NT and T lines were not statistically significant (*F* = 0.9, *p* = 0.35 for I-I; *F* = 1.2, *p* = 0.29 for II-II; and *F* = 0.5, *p* = 0.49 for III-III). Demethylation of CCGG sites due to Al stress (reflected by IV-II, IV-I, and II-I transitions) in NT and T lines equaled 3.75% and 3.97% (Table 4, Fig. 1d, e), respectively. No significant difference in demethylation status was observed between NT and T lines (α = 0.05; *F* = 0.58, *p* = 0.44). De novo methylation (I-IV transition) was observed at 0.23% in two T lines (L195 and L198) (Fig. 1f, Table 4). The observed differences were specific for the two T lines rather than for the whole T set. Moreover, the observed alterations were not significant for NT lines (α = 0.05; *F* = 2.6, *p* = 0.14). No changes in DNA methylation quantitative characteristics of leaves of NT and T lines due to Al stress were observed (not shown).

**Table 4:** Changes in patterns of cytosine methylation in triticale roots under aluminum stress as determined by MSAP

Transitions		Effect	Percentage (%) of the bands representing particular effect											
			NT						T					
			L203	L438	L444	L455	L461	Mean^^^	L195	L198	L201	L210	L451	Mean^^^
IV → I		DM	1.14	2.05	2.05	1.15	1.15	1.74^b^	2.27	1.82	1.81	1.83	2.27	1.73^b^
IV → II		DM	1.60	1.60	1.60	1.84	1.84	1.69^b^	1.59	2.05	1.59	1.83	1.82	1.82^b^
II → I		DM	0.46	0.23	0.23	0.46	0.23	0.32^a^	0.23	0.45	0.45	0.46	0.23	0.41^a^
I → IV		DNM	0	0	0	0	0	0^a^	0.23	0.23	0	0	0	0.09^a^
III → III		LCMS	32.49	32.42	32.42	32.64	32.64	32.45^d^	32.27	32.27	32.20	32.49	32.27	32.38^d^
II → II		LCMS	48.74	48.40	48.40	48.51	48.51	48.45^e^	48.18	47.95	48.53	48.74	48.18	48.29^e^
I → I		LCMS	15.56	15.30	15.30	15.40	15.40	15.36^c^	15.23	15.23	15.42	15.56	15.23	15.28^c^
Total	Demethylation	DM	3.20	3.88	3.88	3.45	3.45	3.75	4.29	4.32	3.85	4.12	4.32	3.97
	Methylation de novo	DNM	0	0	0	0	0	0	0.23	0.23	0	0	0	0.09
	No change	LCMS	96.80	96.12	96.12	96.55	96.55	96.25	95.48	95.45	96.15	95.88	95.68	95.94

*DM*, demethylation event; *DNM*, de novo methylation event; *LCMS*, lack of change in methylation status; *T*, tolerant lines; *NT*, non-tolerant lines

LCMS % = 100 × LCMS / (LCMS + DNM + DM); DNM % = 100 × DNM / (LCMS + DNM + DM); DM % = 100 × DM / (LCMS + DNM + DM)

^^^Means followed by the same letter in the same columns are not significantly different according to Tukey test (α = 0.05)

DNA isolated from roots of NT and T lines grown under control conditions was amplified with 15 selective primer pairs followed by *Acc*65I/*Mse*I and *Kpn*I/*Mse*I digestion, resulting in 567 and 571 reproducible fragments, respectively. On average, approximately 30 and 31 polymorphic fragments were shared between NT and T lines, respectively. The same lines under Al stress allowed for the amplification of 571 (NT) and 568 (T) fragments with 29 and 30 polymorphisms, respectively. Similarly, 571 (NT) and 569 (T) signals were detected when leaves of control and stressed lines were used, with 29 (NT) and 31 (T) polymorphic signals. The majority of polymorphisms were common between roots and leaves of NT and T lines with the exception of two fragments present in the roots of NT lines.

**Fig. 2:**
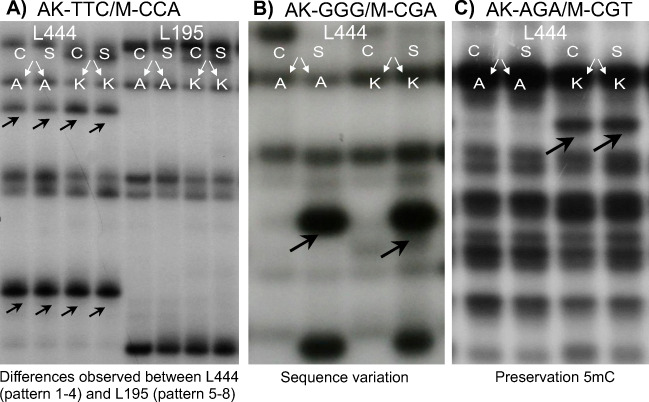
Examples of patterns (denoted by arrows) generated by metAFLP analysis using the following primer combinations: AK-TTC/M-CCA (**a**), differentiation of NT (L444) and T (L195) lines; AK-GGG/M-CGA (**b**), pattern indicating preservation of cytosine methylation; and AK-AGA/M-CGT (**c**), pattern characteristic of sequence variation. *A*, *Acc*65I/*Mse*I digestion; *K*, *Kpn*I/*Mse*I digestion; *C*, control; and *S*, aluminum treatment (20 ppm)

The metAFLP results were converted into four-digit codes. Profiles classified as *1111* were the most frequent in all genotypes for roots, with 536 and 538 profiles identified for T and NT plants, respectively. The number of polymorphisms was 23–43 for T and 32–39 for NT lines, depending on the selective primer pair used. The *0011* pattern was observed 28 and 29 times in roots of five T and five NT lines, respectively. Similarly, for T and NT lines, respectively, the *0100* profile was detected 5 and 4 times; *1010*, 7 and 10 times; *1000*, 3 and 3 times; *1100*, 7 and 11 times; and *0101*, 10 and 11 times. In leaves, the *1111* pattern was identified in 537 cases. The *0011* code was found 26 times in T and NT lines, and *1100* was observed 7 (T) and 11 (NT) times. The *1010*, *1000*, and *0101* profiles were observed exclusively in T lines, with 3, 1, and 4 observations, respectively. Example of metAFLP patterns are shown in Fig. 2.

Quantitative analysis of metAFLP data showed that Al stress elevated the level of sequence variation (SV) in roots of NT and T lines by 0.97% and 0.87%, respectively (Table 5). Patterns reflecting sequence changes were detected at the same loci in both T and NT lines. SV was lower in leaves than in roots and equaled 0.04% and 0.14% for NT and T lines, respectively. Only two T lines (L203 and L451) and one NT line (L461) exhibited sequence changes in leaves, and there were no differences in SV between NT and T lines. However, leaves and roots in T and NT lines differed from one another (Table 5), as indicated by Tukey’s test (α = 0.05; *F* = 12.3, *p* < 0.01). There were no de novo methylation (DNMV) or demethylation (DMV) events in roots and leaves of NT and T plants between control and stressed conditions (Table 5). However, average complex variation, which encompassed simultaneous variation in sequence and methylation, reached 0.24% (NT) and 0.27% (T) in Al-stressed roots. This was not observed in leaves, but the differences between organs were not significant (α = 0.05; *F* = 4.3, *p* > 0.01). Genome methylation (GM) values, reflecting methylation within sequences recognized by the *Acc*65I/*Kpn*I isoschizomers, were approximately 5.0% regardless of organ and line (Table 5). Although differences were not statistically significant, NT plants exhibited slightly higher global methylation levels than T plants in both roots and leaves.

**Table 5:** Mean quantitative metAFLP characteristics for tolerant (T) and non-tolerant (NT) triticale lines after Al^3+^ treatment (20 ppm)

metAFLP characteristics	Abbreviation	Root		Leaf	
		NT (%)	T (%)	NT (%)	T (%)
De novo methylation variation	DNMV	0	0	0	0
Demethylation variation	DMV	0	0	0	0
Genome methylation	GM	5.37^a^	5.10^a^	5.11^a^	4.77^a^
Genome non-methylation	GNM	94.62^a^	94.90^a^	94.89^a^	95.23^a^
Sequence variation	SV	0.97^a^	0.87^a^	0.04^b^	0.14^b^
Complex variation	CV	0.24^a^	0.27^a^	0^a^	0^a^

One-way analysis of variance (ANOVA) with Tukey’s contrasts (α = 0.05) was applied. Means appended with different letters are significantly different

### Sequence analysis of differently methylated loci

Twenty MSAP fragments originating from differentially methylated DNAs extracted from roots of NT and T lines were sequenced. Ten of the fragments reflected demethylation changes and exhibited similarity to annotated sequences in the GenBank (Table 6). The fragments F1, F2, F4, F6, F8, F9, and F10 matched a genomic scaffold located on chromosome 3B in *Triticum aestivum*. Fragments F5 and F7 (Fig. S2) exhibited similarity to the coding region of genes found in *Aegilops tauschii* subsp. *tauschii* encoding receptor-like protein kinase (RLK) and histone-lysine *N*-methyltransferase, respectively. The identified histone-lysine *N*-methyltransferase belonged to the class V-like SAM-binding methyltransferase superfamily. The F3 fragment (Fig. S2) shared similarity with a *Brachypodium distachyon* sequence for peptide chain release factor subunit 1-2-like. The remaining 10 fragments extracted from MSAP gels did not show similarity sequences in the GenBank.

**Table 6:** BLAST results of the DNA methylation polymorphic sequences

Code	Length (bp)	E/HM^a^	Pattern^b^ C → S	Accession	BLAST result
F1	175	AAC/TTC	IV → II	HG670306.1	*Triticum aestivum* chromosome 3B, genomic scaffold, cultivar Chinese Spring
F2	64	AGG/TGA	IV → I	HG670306.1	*Triticum aestivum* chromosome 3B, genomic scaffold, cultivar Chinese Spring
F3	126	ATT/TGT	IV → I	XM_003568528.3	PREDICTED: *Brachypodium distachyon* eukaryotic peptide chain release factor subunit 1-2-like (LOC100821402), mRNA
F4	138	ATT/TGT	IV → I	HG670306.1	*Triticum aestivum* chromosome 3B, genomic scaffold, cultivar Chinese Spring
F5	175	ATT/TGT	IV → II	XM_020340520.1	PREDICTED: *Aegilops tauschii* subsp. *tauschii* putative receptor-like protein kinase At4g00960 (LOC109781929), mRNA
F6	175	ATC/TCA	IV → I	HG670306.1	*Triticum aestivum* chromosome 3B, genomic scaffold, cultivar Chinese Spring
F7	163	ATC/TCA	IV → I	XM_020305332.1	PREDICTED: *Aegilops tauschii* subsp. *tauschii* histone-lysine *N*-methyltransferase, H3 lysine-9 specific SUVH1-like (LOC109746199), mRNA
F8	62	ACA/TGC	IV → I	HG670306.1	*Triticum aestivum* chromosome 3B, genomic scaffold, cultivar Chinese Spring
F9	85	ACA/TGC	IV → I	HG670306.1	*Triticum aestivum* chromosome 3B, genomic scaffold, cultivar Chinese Spring
F10	80	ATT/TCAA	IV → I	HG670306.1	*Triticum aestivum* chromosome 3B, genomic scaffold, cultivar Chinese Spring

*E*, *Eco*RI primers; *HM*, *Hpa*II/*Msp*I primers

^a^Primer combination used in amplification of MSAP fragment

^b^Banding pattern under control (C) and 20 ppm Al^3+^ treatments (S). Band types are referred to Tables 3 and 4

## Discussion

Genetic traits can be readily controlled by plant breeders during selection, but some traits are dependent on environmental factors such as light, temperature, carbon dioxide concentration, humidity, wind, and nutrients (Tsaftaris and Polidoros 2000). Such factors may influence the traits and result in changes even within uniform breeding materials. Molecular approaches capable of identifying markers related to genomic regions affected by epigenetic changes, i.e., due to abiotic stresses, would assist understanding of traits affected by the environment. The plant materials used in this study were highly stable Al-tolerant and Al-sensitive lines developed over years of selective breeding. These fully uniform lines allowed investigation of the Al-tolerant trait.

Al stress is frequently observed in plant populations worldwide. However, no systematic studies devoted to the epigenetic aspects of Al tolerance in crop plants, such as triticale, have been published to date. Epigenetic mechanisms such as DNA methylation and histone alterations play significant roles in protecting organisms from environmental stresses (Mirouze and Paszkowski 2011). DNA methylation triggers the formation of novel epialleles that can be passed to progeny, increasing their ability to adapt to environmental challenges (Iwasaki and Paszkowski 2014). In this study, RP-HPLC, metAFLP, and MSAP analyses were used to assess changes in DNA methylation in Al-tolerant and Al-non-tolerant seedlings exposed to Al. The RP-HPLC and MSAP approaches showed putative alterations in cytosine methylation that were not identified by metAFLP. However, differences identified via the MSAP analysis were not statistically significant, in contrast with those detected by RP-HPLC. MSAP results suggested increased demethylation in the roots of both NT and T lines compared with their non-stressed controls, and no differences were observed between NT and T plants. RP-HPLC analysis identified methylation changes as a result of Al stress in plant roots but not leaves, with a decrease in DNA methylation in NT lines, and an increase in methylation in T lines, observed independent of the Al concentration used. These results are consistent with those of previous studies. Feng et al. (2012) demonstrated that both salt and alkaline stresses triggered demethylation of salt-sensitive genotypes of rice growing for 7 days in stress conditions, while the opposite effect was noticed in salt-tolerant genotypes. Increased DNA methylation in roots was also observed in tolerant rice genotypes under cold stress, but only at the seedling stage (Pan et al. 2011). By contrast, abiotic stresses did not lead to DNA methylation level changes in maize seedings subjected to heat, cold, and UV treatment (Eichten and Springer 2015). These results indicated that stress does not always lead to DNA methylation changes, or that changes were not detectable using the methods employed.

The methylation changes related to Al stress in T and NT triticale genotypes were relatively limited, and only RP-HPLC and to some extent MSAPs were successful in identifying these differences, indicating that a limited portion of the genome responded to Al stress. Al treatment appeared to stimulate DNA methylation changes within specific sequences. This study did not focus on specific sequences, but this suggestion is supported by the MSAP and metAFLP approaches. Genetic mapping using AFLP markers in different species produces non-random distribution of the markers, resulting in clusters and gaps (González et al. 2005). Moreover, sequences recognized by different endonucleases used in distinct AFLP variants may be distributed unevenly with overlapping in various genomic regions. In that context, it may be expected that results would differ between the MSAP and metAFLP analyses. HPLC-RP was able to identify global DNA methylation changes affecting NT and T lines due to Al stress, whereas only a fraction of changes were identified by MSAP. It would be valuable to develop mapping populations to identify the genomic regions affected by methylation changes related to Al stress and determine whether these correspond to Al-tolerance QTLs. This would enhance understanding of the putative epigenetic aspects underlying Al tolerance in triticale.

The metAFLP analysis revealed sequence (SV) changes in T and NT genotypes, particularly in roots and somewhat in leaves. The results indicated the possibly mutagenic action of Al on triticale lines. This analysis is based on the specificity of the *Acc*65I and *Kpn*I isoschizomers. The presence of a band in T (or NT) lines before (or after) and absence of the same band after (or before) Al treatment revealed by *Kpn*I/*Mse*I digests indicates a point mutation event rather than methylation change, as *Kpn*I is insensitive towards site methylation. The direct confirmation of putative mutation could be confirmed via DNA sequencing. However, this is challenging due to the absence of the metAFLP fragment. Sequencing of the respective fragment followed by resequencing of genomic DNA of the control and stressed materials is one approach. However, if the mutation reflects a methylation change then bisulfite sequencing would be needed. Alternatively, the mutagenic action of Al stress could be confirmed from experiments with ISSR markers. Correia et al. (2014) showed that polymorphisms were observed in Al-exposed *Plantago almogravensis* at twice the level in roots than in leaves. Differences between the two organs were not observed in *P. lagopus*, and levels of sequence polymorphism were similar after 7 and 21 days of Al treatment, suggesting that tolerance mechanisms might differ between the two species (Correia et al. 2014). The more frequent alterations seen in triticale roots than in leaves possibly reflect the direct exposure of the roots to Al. Some Al-tolerant plants (excluding ‘Al-accumulating plants’ such as buckwheat and tea (Shen and Ma 2001)) may accumulate toxic compounds in root tissue to prevent dispersal of ions into the other parts of the plant (Fernandes and Henriques 1991). For example, in *Triticum aestivum* L. (Zhang and Taylor 1998) and *Pinus massoniana* (Zhang et al. 2014), Al at concentrations below 200 μM and 1000 μM, respectively, was poorly translocated from roots to leaves and stems. As a result, ions bound in roots affected their structure and growth more than the other parts of the plant (Körpe and Aras 2011). The detailed mechanisms of mutagenic action of Al are not clear, but probably relate to the influence of Al on cellular processes in stressed roots, such as cell division and nucleolus functions (Zhang et al. 2014) or increases in the frequencies of micronuclei formation and anaphase chromosome aberrations in root tips (Yi et al. 2010).

It cannot be excluded that increased SV in NT lines is related to decreased DNA methylation. Alterations in DNA methylation might lead to activation of mobile elements (Iwasaki and Paszkowski 2014; Orłowska et al. 2016), and these could act as a source of mutations (Saze et al. 2012). In maize, silent transposable elements (TEs) were hypermethylated whereas active elements were hypomethylated (Banks et al. 1998; Chandler and Walbot 1986). A similar investigation in rice showed that the transpositional activity of Tos17 retrotransposon was negatively associated with the level of DNA methylation (Cheng et al. 2006). Thus, it is possible that increased DNA methylation due to Al stress in T lines resulted in decreased SV as the consequence of the better stability of TEs. Previous studies showed that DNA methylation levels are closely related to the regulation of gene expression (Peng and Zhang 2009). The role of DNA methylation changes due to Al stress was demonstrated in tobacco (Choi and Sano 2007). In that case, demethylation affected the coding region of the *NtGPDL* gene, which encoded a glycerophosphodiesterase-like protein and was linked to the response to Al, paraquat, salt, and cold stresses in tobacco, resulting in enhanced expression of the gene (Choi and Sano 2007). Similarly, the majority of MSAP fragments identified in this study also reflected demethylation at least in the region of the restriction site. Three of the sequences showed similarity to the coding regions of genes potentially involved in stress responses. Of these, RLK can sense environmental changes and then transduce this information via activated signaling pathways to trigger adaptive responses (Morris and Walker 2003). Histone-lysine *N*-methyltransferase and H3 lysine-9-specific SUVH1-like catalyze the transfer of methyl groups to lysine and arginine residues of histone proteins (Thorstensen et al. 2011). Such epigenetic modification is crucial for regulation of chromatin structure and gene expression in eukaryotes. The identification of a MSAP fragment with sequences exhibiting similarity to the peptide chain release factor subunit 1-2-like gene could be related to the destructive impact of Al on root growth. This gene is involved in growth regulation and the termination of protein synthesis (Zhouravleva et al. 1995), and mutations within the gene sequence can lead to the reduction of internode spacing and growth inhibition (Petsch et al. 2005). It was not possible to identify sequences with significant similarity for other MSAP markers, probably as a result of their short sequences.

## Conclusions

This study showed that exposure to Al^3+^ at concentrations of 15, 20, and 30 ppm induced genome-wide changes in DNA methylation/demethylation in triticale roots but not in leaves. Al, a toxic and mutagenic metal, also likely contributed to sequence changes observed primarily in leaves but also to a smaller extent in leaves. It was possible that the mutagenic action of Al^3+^ was related to activation of TEs due to DNA demethylation observed in NT lines. Further investigation is needed to fully understand the impact of epigenetic DNA methylation changes in response to Al stress in roots of T and NT triticale lines.

## Electronic supplementary material

Table S1 (DOCX 14 kb)

Table S2 (DOCX 14 kb)

Fig. S1 Methylation patterns in roots and leaves of triticale lines (tolerant L198 and non-tolerant L444) treated with 15 and 30 ppm Al^3+^. Examples show patterns for the following primer combinations: HM-TCG/E-AGA (**a**), HM-TGA/E-AGG (**b**), HM-TGC/E-ACA (**c**), and HM-TCAA/E-ACC (**d**). *H*, *Hpa*II/*Eco*RI digestion; *M*, *Msp*I/*Eco*RI digestion; *C*, control. S15 and S30 indicate aluminum in concentrations of 15 and 30 ppm, respectively. (PDF 168 kb)

Fig. S2 **a**, **b** MSAP fragments exhibited homology with the coding region of peptide chain release factor subunit 1-2-like (F3), receptor-like protein kinase (F5), and histone-lysine *N*-methyltransferase (F7) (fragment names according to the Table 6). (PDF 76 kb)
