# Supplementary material for: The influence of Al3+ on DNA methylation and sequence changes in the triticale (× Triticosecale Wittmack) genome
Source: J Appl Genet. 2018 Aug 30;59(4):405–17. doi: 10.1007/s13353-018-0459-0 (PMC7902597; doi:10.1007/s13353-018-0459-0)
Supplement: Supplementary file 1 — (DOCX 14 kb) [file 13353_2018_459_MOESM1_ESM.docx]

Additional file 1: Table S1. Adapter and primer sequences for metAFLP analysis.

|  | *Mse*I | *Acc65*I/*Kpn*I |
| --- | --- | --- |
| Adapter1 | 5'-TACTCAGGACTCATC-3' | *Acc*65I 5'-CTCGTAGCATGCGTACA-3' |
|  |  | *Kpn*I 5'-CTCGTAGCATGCGTACAGTAC-3' |
| Adapter2 | 5'-GAGTCCTGAGTAGCAG-3' | *Acc*65I 5'-GTACTGTACGCATGCTAC-3' |
|  |  | *Kpn*I 5'-ACTCGTACGACTGT-3' |
| Pre-amp primer | 5'- GATGAGTCCTGAGTAAC-'(M1) | 5'- G CAT GCG TAC AGT ACC-3' |
|  |  | 5'- CAT GCG TAC AGT ACC-3'(AK1) |
| Selective primer | M1+GT (M01) | AK1+AGA (AK01) |
|  | M1+AT (M02) | AK1+TAG (AK02) |
|  | M1+CG (M03) | AK1+TTG (AK03) |
|  | M1+TG (M04) | AK1+AGG (AK04) |
|  | M1+AT (M05) | AK1+AGC (AK05) |
|  | M1+GA (M06) | AK1+GGG (AK06) |
|  | M1+GG (M07) | AK1+ACG (AK07) |
|  | M1+CT (M08) | AK1+GTA (AK08) |
|  | M1+GC (M09) | AK1+GAG (AK09) |
|  | M1+TA (M10) | AK1+TCG (AK10) |
|  | M1+TT (M11) | AK1+TTC (AK11) |
|  | M1+CC (M12) | AK1+AAA (AK12) |
|  | M1+AA (M13) | AK1+TAC (AK13) |
|  | M1+AG (M14) | AK1+AAT (AK14) |
|  | E1+CA (E15) | AK1+TTA (AK15) |
