# Supplementary material for: The influence of Al3+ on DNA methylation and sequence changes in the triticale (× Triticosecale Wittmack) genome
Source: J Appl Genet. 2018 Aug 30;59(4):405–17. doi: 10.1007/s13353-018-0459-0 (PMC7902597; doi:10.1007/s13353-018-0459-0)
Supplement: Supplementary file 2 — (DOCX 14 kb) [file 13353_2018_459_MOESM2_ESM.docx]

Additional file 2: Table S2. Adapter and primer sequences for MSAP analysis.

|  | *Eco*RI | *Msp*I/*Hpa*II |
| --- | --- | --- |
| Adapter1 | 5'-CTCGTAGACTGCGTACC-3' | 5'- GATCATGAGTCCTGCT-3' |
| Adapter2 | 5'-AATTGGTACGCAGTCTAC-3' | 5'- CGAGCAGGACTCATGA-3' |
| Pre-amp primer | 5'-GACTGCGTACCAATTCA-3'(E1) | 5'- ATCATGAGTCCTGCTCGG-3'(HM1) |
| Selective primer | E1+CG (E01) | HM1+TTG (HM01) |
|  | E1+AC (E02) | HM1+TTC (HM02) |
|  | E1+GG (E03) | HM1+TGA (HM03) |
|  | E1+CT (E04) | HM1+TCG (HM04) |
|  | E1+GT (E05) | HM1+TGC (HM05) |
|  | E1+AG (E06) | HM1+TAC (HM06) |
|  | E1+TT (E07) | HM1+TGT (HM07) |
|  | E1+TC (E08) | HM1+TCA (HM08) |
|  | E1+GA (E09) | HM1+TCG (HM09) |
|  | E1+CA (E10) | HM1+TGC (HM10) |
|  | E1+GC (E11) | HM1+TTGC (HM11) |
|  | E1+CC (E12) | HM1+TCAA (HM12) |
|  | E1+TG (E13) | HM1+TCAA (HM13) |
|  | E1+TT (E14) | HM1+TCAA (HM14) |
