# Supplementary figures and images for: The influence of Al3+ on DNA methylation and sequence changes in the triticale (× Triticosecale Wittmack) genome
Source: J Appl Genet. 2018 Aug 30;59(4):405–17. doi: 10.1007/s13353-018-0459-0 (PMC7902597; doi:10.1007/s13353-018-0459-0)

**A) HM-TCG/E-AGA**

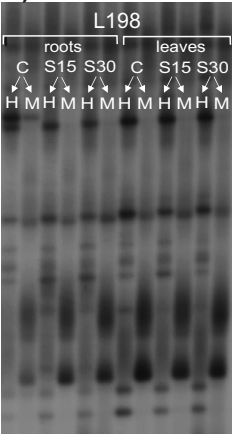

**B) HM-TGA/E-AGG**

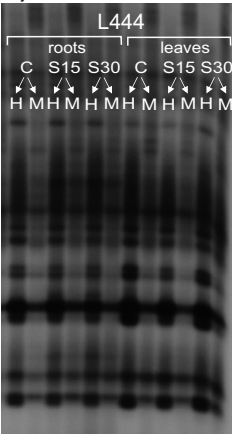

**D) HM-TGC/E-ACA**

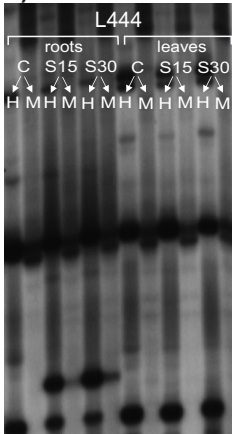

**C) HM-TCAA/E-ACC**

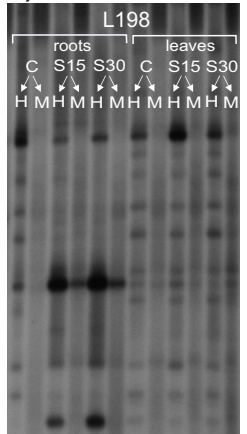

Supplement: Supplementary file 3 — Methylation patterns in roots and leaves of triticale lines (tolerant L198 and non-tolerant L444) treated with 15 and 30 ppm Al3+. Examples show patterns for the following primer combinations: HM-TCG/E-AGA (a), HM-TGA/E-AGG (b), HM-TGC/E-ACA (c), and HM-TCAA/E-ACC (d). H, HpaII/EcoRI digestion; M, MspI/EcoRI digestion; C, control. S15 and S30 indicate aluminum in concentrations of 15 and 30 ppm, respectively. (PDF 168 kb) [file 13353_2018_459_MOESM3_ESM.pdf]

**A) HM-TCA/E-ATC** **B) HM-TGT/E-ATT**

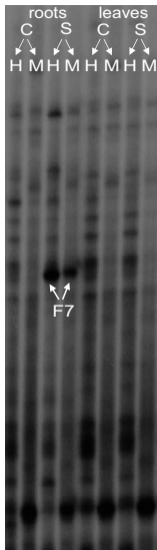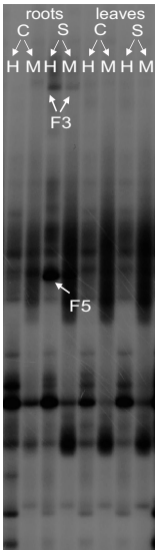

Supplement: Supplementary file 4 — a, b MSAP fragments exhibited homology with the coding region of peptide chain release factor subunit 1-2-like (F3), receptor-like protein kinase (F5), and histone-lysine N-methyltransferase (F7) (fragment names according to the Table 6). (PDF 76 kb) [file 13353_2018_459_MOESM4_ESM.pdf]
